# Parental smoking and childhood cancer: results from the United Kingdom Childhood Cancer Study

**DOI:** 10.1038/sj.bjc.6600774

**Published:** 2003-02-10

**Authors:** D Pang, R McNally, J M Birch

**Affiliations:** 1 Cancer Research UK, Paediatric and Familial Cancer Research Group, Royal Manchester Children's Hospital, Stancliffe, Hospital Road, Manchester M27 4HA, UK

**Keywords:** case – control study, childhood cancer, hepatoblastoma, parental smoking

## Abstract

There are strong *a priori* reasons for considering parental smoking behaviour as a risk factor for childhood cancer but case – control studies have found relative risks of mostly only just above one. To investigate this further, self-reported smoking habits in parents of 3838 children with cancer and 7629 control children included in the United Kingdom Childhood Cancer Study (UKCCS) were analysed. Separate analyses were performed for four major groups (leukaemia, lymphoma, central nervous system tumours and other solid tumours) and more detailed diagnostic subgroups by logistic regression. In the four major groups, after adjustment for parental age and deprivation there were nonsignificant trends of increasing risk with number of cigarettes smoked for paternal preconception smoking and nonsignificant trends of decreasing risk for maternal preconception smoking (all *P*-values for trend >0.05). Among the diagnostic subgroups, a statistically significant increased risk of developing hepatoblastoma was found in children whose mothers smoked preconceptionally (OR=2.68, *P*=0.02) and strongest (relative to neither parent smoking) for both parents smoking (OR=4.74, *P*=0.003). This could be a chance result arising from multiple subgroup analysis. Statistically significant negative trends were found for maternal smoking during pregnancy for all diagnoses together (*P*<0.001) and for most individual groups, but there was evidence of under-reporting of smoking by case mothers. In conclusion, the UKCCS does not provide significant evidence that parental smoking is a risk factor for any of the major groups of childhood cancers.

There are strong *a priori* reasons for considering parental smoking behaviour as a risk factor for childhood cancer. Many proven carcinogens are present in tobacco and tobacco smoke (Hecht, 1999; Hoffmann and Hoffmann, 1997). Tobacco-related compounds have also been detected in human placenta, fetal blood, urine of offspring and in breast milk of smoking mothers (Perlman and Dannenberg, 1942; Everson *et al*, 1986,^1988^; Woodward *et al*, 1986; Hansen *et al*, 1992; Myers *et al*, 1996; Pinorini-Godly and Myers, 1996; Arnould *et al*, 1997; Daube *et al*, 1997).

Previous case–control studies have tended to show weak associations between maternal cigarette smoking during pregnancy and childhood cancers (IARC, 1986; Severson *et al*, 1993; Tredaniel *et al*, 1994; Sorahan *et al*, 1995,1997a,1997b,^2001^; Klebanoff *et al*, 1996; Shu *et al*, 1996). There is somewhat stronger and more consistent evidence for a paternal preconceptional effect, although relative risks in most case–control studies are only just above one (Severson *et al*, 1993; Shu *et al*, 1996; Ji *et al*, 1997; Sorahan *et al*, 1997b; 2001). This effect has been attributed to germ-cell mutations during spermatogenesis caused by tobacco products (Wyrobeck, 1993; Wyrobek and Adler, 1996; Woodall and Ames, 1997). Relevant studies are reviewed by Thornton and Lee (1998).

The United Kingdom Childhood Cancer Study (UKCCS) is a nationwide population-based case–control study of possible aetiological factors in childhood cancer. We have examined the possible effect of paternal and maternal cigarette smoking in the development of childhood cancers by analysing self-reported parental smoking habits in relation to such cancers at a preconceptional time period and maternal smoking during pregnancy. Information on paternal smoking during pregnancy was not collected as maternal passive smoking during pregnancy was not considered important when the study was designed.

## MATERIALS AND METHODS

The UKCCS study design, case and control selection, and data collection procedures have already been published in detail (McKinney *et al*, 1995; UK Childhood Cancer Study Investigators, 2000). A summary is provided here.

### Case ascertainment

The study sought to interview the parents of children, resident in England, Scotland and Wales, who were diagnosed with a confirmed malignancy or any central nervous system (CNS) tumour under the age of 15 years between 1991–1994 in Scotland and 1992–1994 in England and Wales. Case accrual continued in England and Wales for non-Hodgkin's lymphoma and leukaemias during 1995 and leukaemias alone during 1996. A total of 10 study regions were defined. Cases were usually ascertained from regional paediatric oncology units. Completeness of ascertainment was checked by cross-reference to regional and national cancer registries. Leukaemia diagnoses and subclassification were based on all available data including data from national clinical trials. For solid tumours, consensus diagnoses were made by panels of pathologists with special interests in specific types of tumours after histopathological review. All diagnoses were coded according to *International Classification of Diseases for Oncology* (2nd ed.) (Percy *et al*, 1990) and regrouped into Birch and Kelsey diagnostic subcategories (UKCCS Investigators, 2000), available at http://www.biomed2.man.ac.uk/crcpfcrg/crukpfcrg/pfcrg.htm.

### Control selection

For each case child, similar data were sought for two control children matched for sex, date of birth and geographical area of residence at diagnosis and randomly selected from Family Health Services Authorities (FHSA) lists in England and Wales and Health Boards in Scotland. Control parents were contacted following permission from their general practitioners (GP). The parents of 3838 case children and 7629 control children were interviewed, representing participation rates of 87 and 64%, respectively.

### Parental smoking behaviour

Evaluable parental smoking data obtained from face-to-face structured interviews with parents were available on 3585 case fathers, 6987 control fathers, 3814 case mothers and 7581 control mothers. We developed software to derive the preconception smoking variable from the social habits section of the questionnaire on the basis of the starting and stopping dates of smoking, specific questions relating to smoking 1 year before birth of the index child and at other time points and the birth date of the index child. Parental smoking status was categorised as: (a) lifelong nonsmokers; (b) ex-smokers, who stopped smoking more than 1 year before the birth of the index child; and (c) current smokers, who smoked cigarettes during the year before the birth of the index child. When smoking status was unknown (8% of parents overall) because of missing information or contradictory information, these were excluded from statistical analyses to avoid bias of odds ratios (ORs) because of possible misclassification of smoking status (Lee, 1998; Infante-Rivard and Jacques, 2000). A smoking variable defining numbers of cigarettes smoked per day during specific periods of pregnancy was derived from the interview data. Maternal smoking during the second trimester of pregnancy is reported since fetal organs have by then developed and have begun to function. It was considered that fetal organ exposure to products of tobacco smoking in this period might replicate the tissue-specific effects suggested by animal studies.

### Statistical methods

As in previous UKCCS reports (UKCCS Investigators, 1999; Beral *et al*, 2001), case and control data relating to cigarette consumption were compared by means of unconditional logistic regression (Breslow and Day, 1980) using Stata, version 6, program (StataCorp, 1999). The original matching variables were accounted for by adjustment, with all analyses being routinely adjusted for child's age at diagnosis in single years (0–14) treated as a continuous variable, sex and UKCCS region (Beral *et al*, 2001). All controls were used as the comparison group for each diagnostic group. Since it is known that spontaneous mutation rates can increase with age, especially in males (Penrose, 1955,^1957^; Vogel and Rathenberg, 1975), adjustments were made for parental ages at the birth of the child (in single years) and for deprivation score. The latter is a small-area seven-level index based on car ownership, overcrowding and unemployment. Individual scores were obtained by linking to the 1991 census of Great Britain via the postcodes of their residence at diagnosis/pseudodiagnosis of the index children (UKCCS Investigators, 2000).

Analyses were performed with respect to paternal and maternal preconception smoking, and maternal smoking in pregnancy, initially by the following broad diagnostic groups: all cancers, leukaemia, lymphoma, central nervous system tumours (CNS) and other solid tumours. Possible dose–response relations between smoking and childhood cancer were examined using three smoking categories of 0, 1–19, and 20 or more cigarettes smoked per day (cpd). Relative risks were estimated for light/moderate (1–19 cpd) and heavy (20+ cpd) smokers relative to nonsmokers. ORs were computed and shown relative to a baseline risk of unity for the nonsmokers together with two-sided *P*-values and 95% confidence intervals (CI). The trend of risk by amount smoked was examined by coding the three smoking groups to 1, 2 and 3, respectively, and treating as a continuous variable (Sorahan *et al*, 1997a,1997b).

Work on single gene cancer predisposition syndromes, on specific somatic mutations in human cancers and on laboratory animals suggests that any mutagenic/carcinogenic effects of tobacco smoke would be tissue specific and/or organ specific (Correa *et al*, 1990; Beebe *et al*, 1993; Hecht, 1998; Birch, 1999; Hollstein *et al*, 1999). Therefore, separate analyses were performed for Birch and Kelsey diagnostic subgroups, which group biologically similar tumours together (UKCCS Investigators, 2000). In those groups showing a statistically significantly elevated OR, the relation was explored in more detail as follows: father's smoking in the absence of mother's smoking, mother's smoking in the absence of father's smoking, and both father and mother smoking were compared with neither smoking for data on parental preconception smoking.

If parental smoking is a risk factor for childhood cancer, germline mutations caused by a preconceptional exposure might lead to earlier age of cancer onset in the child, whereas a transplacental exposure may be associated with later age of onset. To investigate this possibility, analyses were carried out for children, under and above the median age at diagnosis, respectively, in diagnostic groups with elevated ORs.

## RESULTS

Table 1

**Table 1:** Characteristics of study subjects and their biological parents

	**Maternal data available**				**Paternal data available**			
	**Cases**		**Controls**		**Cases**		**Controls**	
	***n***	**%**	***n***	**%**	***n***	**%**	***n***	**%**
*Child*								
Sex								
Boys	2139	56.1	4259	56.2	2020	56.3	3913	56.0
Girls	1675	43.9	3322	43.8	1565	43.7	3074	44.0
Age at diagnosis (years)								
0	315	8.3	632	8.3	307	8.6	604	8.6
1–4	1596	41.8	3175	41.9	1515	42.3	2943	42.1
5–9	1042	27.3	2057	27.1	971	27.1	1880	26.9
10–14	861	22.6	1717	22.6	792	22.1	1560	22.3
								
*Parent*								
Age at birth of the child (years)								
Mean (s.d.)	27.5 (5.2)		27.9 (5.2)		30.4 (6.1)		30.7 (6.0)	
Median	27.3		27.7		29.8		30.1	
Parental cigarette smoking at conception								
No	2344	61.5	4674	61.7	1964	54.8	3909	55.9
Yes	1193	31.3	2375	31.3	1348	37.6	2463	35.3
Unknown	277	7.3	532	7.0	273	7.6	615	8.8
Parental cigarette smoking in pregnancy								
No	2958	77.6	5743	75.8	—	—	—	—
Yes	855	22.4	1834	24.2	—	—	—	—
Unknown	1	0.0	4	0.0	—	—	—	—

shows details of subjects and their biological parents. In total 56% are boys and about 50% are under 5 years old at diagnosis. Case and control children have similar distributions by sex and age at diagnosis. The mother's mean age at birth of the index child is 27 years and the father's is 30 years. Case parents are slightly younger than control parents. For all cancers, ORs for the child's age at diagnosis and sex are close to 1 due to matching (all ORs=1.0, all *P*-values⩾0.5). Nevertheless, these matching variables were included in the model to avoid a biased estimate of the parental smoking effect (Clayton and Hills, 1993). There is a significant negative trend for maternal age at the birth of the index child (*P*-value for trend <0.001), but a nonsignificant positive trend for paternal age (*P*-value for trend=0.4). There is a significant positive trend for deprivation (*P*-value for trend <0.05).

Table 2

**Table 2:** Paternal preconception cigarette smoking in relation to childhood cancer risks

			**Before adjustment**				**After adjustment^a^**			
**Diagnostic group**	**Cases**	**Controls**	**OR**	**95% CI**			**OR**	**95% CI**		
*All cancer*	3585	6987								
Lifelong nonsmoker	1543	3082	1.00				1.00			
1–19 cpd	583	1003	1.16*	1.03	1.31		1.11	0.98	1.25	
20+ cpd	757	1440	1.05	0.94	1.17		1.01	0.90	1.12	
Trend *P*						0.198				0.635
Ex-smoker	421	827	1.02	0.89	1.16		1.05	0.92	1.20	
Smoking status n/k	281	635								
										
*Leukaemia*	1630	6987								
Lifelong nonsmoker	697	3082	1.00				1.00			
1–19 cpd	269	1003	1.19*	1.01	1.39		1.12	0.96	1.32	
20+ cpd	342	1440	1.07	0.93	1.24		1.01	0.87	1.17	
Trend *P*						0.216				0.743
Ex-smoker	192	827	1.03	0.86	1.23		1.05	0.87	1.25	
Smoking status n/k	130	635								
										
*Lymphoma*	331	6987								
Lifelong nonsmoker	124	3082	1.00				1.00			
1–19 cpd	55	1003	1.34	0.96	1.87		1.27	0.91	1.70	
20+ cpd	72	1440	1.15	0.85	1.56		1.09	0.80	1.49	
Trend *P*						0.260				0.419
Ex-smoker	45	827	1.32	0.93	1.89		1.43	0.99	2.05	
Smoking status n/k	35	635								
										
*CNS*	635	6987								
Lifelong nonsmoker	271	3082	1.00				1.00			
1–19 cpd	101	1003	1.13	0.89	1.43		1.08	0.85	1.38	
20+ cpd	138	1440	1.07	0.86	1.33		1.03	0.82	1.28	
Trend *P*						0.466				0.706
Ex-smoker	71	827	0.98	0.74	1.28		1.01	0.76	1.33	
Smoking status n/k	54	635								
										
*Other solid tumours*	989	6987								
Lifelong nonsmoker	451	3082	1.00				1.00			
1–19 cpd	158	1003	1.08	0.89	1.31		1.05	0.86	1.28	
20+ cpd	205	1440	0.99	0.83	1.18		0.98	0.81	1.17	
Trend *P*						0.981				0.909
Ex-smoker	113	827	0.95	0.76	1.19		1.01	0.81	1.26	
Smoking status n/k	62	635								

* *P*<0.05.

a For deprivation and parental age at birth of index child. cpd=cigarettes per day, n/k=not known.

shows ORs for paternal preconception smoking for the main diagnostic groups. Overall, there was a nonsignificant trend of increasing risks with amount smoked after adjustment for parental age and deprivation. Although some statistically significant ORs were shown among fathers with 1–19 cpd, adjustment for parental age and deprivation removed these.

For maternal preconception smoking, Table 3

**Table 3:** Maternal preconception cigarette smoking in relation to childhood cancer risks

			**Before adjustment**				**After adjustment^a^**			
**Diagnostic group**	**Cases**	**Controls**	**OR**	**95% CI**			**OR**	**95% CI**		
*All cancer*	3814	7581								
Lifelong nonsmoker	1991	3916	1.00				1.00			
1–19 cpd	795	1490	1.05	0.95	1.16		1.00	0.90	1.11	
20+ cpd	394	882	0.88	0.77	1.00		0.86*	0.75	0.99	
Trend *P*						[0.206]				[0.072]
Ex-smoker	353	758	0.92	0.80	1.05		0.95	0.83	1.10	
Smoking status n/k	281	535								
										
*Leukaemia*	1723	7581								
Lifelong nonsmoker	899	3916	1.00				1.00			
1–19 cpd	351	1490	1.04	0.90	1.19		0.99	0.86	1.15	
20+ cpd	191	882	0.96	0.81	1.14		0.96	0.80	1.16	
Trend *P*						[0.864]				[0.652]
Ex-smoker	153	758	0.86	0.71	1.04		0.88	0.73	1.07	
Smoking status n/k	129	535								
										
*Lymphoma*	351	7581								
Lifelong nonsmoker	180	3916	1.00				1.00			
1–19 cpd	67	1490	0.95	0.71	1.27		0.87	0.63	1.18	
20+ cpd	38	882	0.93	0.65	1.34		0.86	0.58	1.27	
Trend *P*						[0.624]				[0.274]
Ex-smoker	39	758	1.15	0.80	1.65		1.25	0.86	1.80	
Smoking status n/k	27	535								
										
*CNS*	684	7581								
Lifelong nonsmoker	349	3916	1.00				1.00			
1–19 cpd	143	1490	1.06	0.86	1.30		1.00	0.80	1.24	
20+ cpd	67	882	0.84	0.64	1.10		0.78	0.58	1.05	
Trend *P*						[0.385]				[0.157]
Ex-smoker	67	758	1.01	0.77	1.33		1.05	0.79	1.39	
Smoking status n/k	58	535								
										
*Other solid tumours*	1056	7581								
Lifelong nonsmoker	563	3916	1.00				1.00			
1–19 cpd	234	1490	1.09	0.93	1.29		1.05	0.88	1.25	
20+ cpd	98	882	0.77*	0.61	0.96		0.75*	0.58	0.96	
Trend *P*						[0.144]				[0.111]
Ex-smoker	94	758	0.86	0.68	1.09		0.92	0.73	1.16	
Smoking status n/k	67	535								

* For deprivation and parental age at the birth of the index child.

a *P*<0.05. cpd=cigarettes per day. [ ] indicates negative trend, n/k=not known.

shows a statistically significant decreased OR of 0.86 among heavy smokers after adjustment (*P*=0.03), also seen for other solid tumours (OR=0.75, *P*=0.02).

For all diagnoses considered together, the OR for paternal preconceptional smoking in cases below, or at or above, the median age was 1.03 (*P*=0.7) and 1.05 (*P*=0.4), respectively. For maternal preconceptional smoking, the corresponding ORs were 0.91 (*P*=0.2) and 1.00 (*P*=1.0). Therefore, there was no evidence of a general tendency for increased risk at younger ages that may have been associated with germline mutations in relevant genes.

Table 4

**Table 4:** Maternal cigarette smoking during pregnancy in relation to childhood cancer risks

			**Before adjustment**				**After adjustment^a^**			
**Diagnostic group**	**Cases**	**Controls**	**OR**		**95% CI**		**OR**		**95% CI**	
*All cancer*										
Nonsmoker	2958	5743	1.00				1.00			
1–19 cpd	648	1306	0.96	0.87	1.06		0.92	0.82	1.03	
20+ cpd	207	528	0.76**	0.64	0.90		0.71***	0.59	0.85	
Trend *P*						[0.004]				[<0.001]
*Leukaemia*										
Nonsmoker	1341	5743	1.00				1.00			
1–19 cpd	286	1306	0.95	0.83	1.10		0.93	0.80	1.08	
20+ cpd	95	528	0.80	0.64	1.01		0.76*	0.60	0.98	
Trend *P*						[0.069]				[0.029]
*Lymphoma*										
Nonsmoker	269	5743	1.00				1.00			
1–19 cpd	60	1306	0.99	0.74	1.32		0.92	0.67	1.26	
20+ cpd	22	528	0.82	0.52	1.29		0.72	0.44	1.20	
Trend *P*						[0.481]				[0.208]
*CNS*										
Nonsmoker	538	5743	1.00				1.00			
1–19 cpd	111	1306	0.89	0.72	1.11		0.86	0.68	1.08	
20+ cpd	35	528	0.69*	0.48	0.98		0.62*	0.42	0.93	
Trend *P*						[0.029]				[0.011]
*Other solid tumours*										
Nonsmoker	810	5743	1.00				1.00			
1–19 cpd	191	1306	1.02	0.86	1.21		0.94	0.78	1.13	
20+ cpd	55	528	0.73*	0.55	0.97		0.68*	0.49	0.93	
Trend *P*						[0.123]				[0.030]

a For parental age and deprivation.

* *P*<0.05;

** *P*<0.01;

*** *P*<0.001. cpd=cigarettes per day. [ ] indicates negative trend.

shows, for maternal smoking during pregnancy, significant monotonic decreasing trends in risk with amount smoked for all cancers, leukaemia, lymphoma, CNS tumours and other solid tumours (*P*<0.001, *P*=0.03, *P*=0.01 and *P*=0.03 respectively), with ORs statistically significantly below 1 among heavy smokers.

Table 5

**Table 5:** Parental smoking in relation to childhood cancer risks by selected diagnostic subgroups

	**Father preconception**				**Mother preconception**				**Mother pregnancy**			
**Diagnostic group^a^**	**Cases^b^**	**OR^d^**	**95% CI**		**Cases^c^**	**OR^d^**	**95% CI**		**Cases^c^**	**OR^d^**	**95% CI**	
110 Acute lymphocytic leukaemia	1375	1.04	0.91	1.18	1449	1.02	0.89	1.16	1449	0.89	0.77	1.03
120 Acute myeloid leukaemia	230	1.07	0.80	1.43	249	0.82	0.60	1.12	249	0.76	0.54	1.07
130 Chronic myeloid leukaemia	22	1.44	0.59	3.50	22	1.44	0.57	3.65	22	1.70	0.68	4.26
210 Hodgkin's disease	105	1.16	0.75	1.70	114	0.75	0.47	1.19	114	0.91	0.57	1.45
220 Non-Hodgkin's lymphoma	218	1.03	0.76	1.40	229	0.90	0.65	1.23	229	0.86	0.61	1.22
311 Pilocytic astrocytoma	158	1.08	0.76	1.52	169	0.89	0.62	1.28	169	0.92	0.62	1.37
315 Other astrocytoma	119	0.92	0.61	1.39	132	0.94	0.62	1.43	132	0.84	0.53	1.33
320 Other glioma	66	1.09	0.64	1.84	67	0.97	0.55	1.71	67	1.12	0.63	1.99
330 Ependymoma	61	1.03	0.59	1.78	69	0.73	0.40	1.35	69	0.77	0.40	1.47
340 Choroid plexus tumours	20	2.11	0.81	5.49	20	1.29	0.44	3.79	20	0.49	0.14	1.75
350 Primitive neuroectodermal tumours	150	0.90	0.63	1.30	161	0.96	0.65	1.42	161	0.55*	0.34	0.88
360 Mis. brain and spinal neoplasms	61	1.33	0.77	2.28	66	0.82	0.46	1.45	66	0.74	0.39	1.40
400 Retinoblastoma	86	0.66	0.40	1.09	87	0.60	0.40	1.10	87	0.60	0.30	1.10
510 Neuroblastoma	180	1.35	0.97	1.88	188	1.04	0.73	1.49	188	0.91	0.62	1.34
520 Peripheral neuroectodermal tumours	74	1.12	0.68	1.80	78	1.52	0.93	2.48	78	1.48	0.88	2.47
610 Wilms' tumour	170	1.01	0.73	1.42	182	0.82	0.57	1.17	182	0.82	0.55	1.22
630 Other and unspecified renal tumours	10	1.76	0.37	8.31	12	0.98	0.26	3.71	12	0.98	0.24	3.99
710 Hepatoblastoma	27	2.19	0.94	5.12	28	2.68*	1.16	6.21	28	1.10	0.44	2.72
810 Osteosarcoma	48	1.18	0.65	2.14	56	1.06	0.56	2.01	56	0.46	0.20	1.06
830 Rhabdomyosarcoma	125	0.84	0.57	1.25	132	0.83	0.55	1.25	132	1.02	0.66	1.56
850 Other soft tissue sarcoma	62	0.81	0.46	1.43	69	1.02	0.57	1.80	69	0.98	0.53	1.83
910 Gonadal germ cell tumours	34	0.98	0.47	2.03	35	0.82	0.37	1.84	35	0.70	0.28	1.75
920 Nongonadal germ cell tumours	57	0.81	0.46	1.46	61	0.74	0.41	1.37	61	0.53	0.25	1.10
1000 Rare miscellaneous tumours	51	1.14	0.62	2.08	57	1.21	0.66	2.22	57	1.16	0.62	2.18
1200 Langerhans cell histiocytosis	46	0.73	0.38	1.40	49	0.74	0.37	1.48	49	0.47	0.20	1.15

a Birch–Kelsey classification (UKCCS Investigators, 2000), excluding diagnostic subgroups with less than 10 cases.

b Corresponding controls: 6987.

c Corresponding controls: 7581.

d After adjustment for parental age and deprivation.

* *P*<0.05.

shows ORs by the Birch–Kelsey diagnostic subgroup. For paternal preconception smoking, most subgroups showed ORs close to unity, but choroid plexus tumours and hepatoblastoma showed statistically nonsignificant elevated ORs (OR=2.1 and 2.2) after adjustment for parental age and deprivation. For maternal preconception smoking, only hepatoblastoma showed a significantly elevated OR (OR=2.68, *P*=0.02) after adjustment for parental age and deprivation. For the other subgroups, ORs were close to unity. For maternal smoking during pregnancy, ORs in most diagnostic subgroups were below unity and not statistically significant, but for primitive neuroectodermal tumours the OR was 0.55 (*P*=0.01).

For hepatoblastoma, Table 6

**Table 6:** Parental smoking in relation to risk of developing hepatoblastoma (all results adjusted for parental age and deprivation)

	**Father preconception**						**Mother preconception**						**Mother 2nd trimester**					
	**Cases**	**Controls**	**OR**	**95% CI**			**Cases**	**Controls**	**OR**	**95% CI**			**Cases**	**Controls**	**OR**	**95% CI**		
*Total*	27	6987	2.19	0.94	5.12		28	7581	2.68*	1.16	6.21		28	7581	1.10	0.43	2.41	
Age of the index child																		
Age< median	13	823	1.28	0.37	4.41		14	897	0.71	0.18	2.84		14	896	0.92	0.49	2.89	
⩾median	14	6164	3.27	0.94	11.33		14	6684	12.02**	2.54	56.75		14	6685	1.28	0.44	2.72	
																		
No. of cigarattes smoked per day																		
0	11	3082	1.00				10	3916	1.00				21	5743	1.00			
1–19	6	1003	1.88	0.67	5.26		9	1490	2.99*	1.15	7.76		5	1306	1.09	0.39	3.04	
20+	7	1440	1.65	0.61	4.45		4	882	2.17	0.65	7.20		2	528	1.12	0.25	4.99	
Trend *P*						0.272						0.058						0.844
Ex-smoker	0	827					2	758	0.98	0.21	4.53							
																		
Parental smoking																		
Neither parent	8	3142	1.00															
Mother only	2	574	2.02	0.40	10.20													
Father only	3	1008	1.86	0.46	7.55													
Both parents	10	1249	4.74**	1.68	13.35													

* *P*<0.05.

** *P*<0.01.

shows results for parental smoking by age, number of cpd, and number of parents smoking. For maternal preconception smoking, there was a statistically significantly elevated OR of 12 for children older than the median age at diagnosis after adjustment for parental age and deprivation (*P*=0.002) and a positive trend with number of cigarettes smoked per day of borderline statistical significance (*P*=0.06). The strongest effect is seen for both parents smoking preconceptionally (OR=4.74, *P*=0.003).

## DISCUSSION

The UKCCS does not provide evidence that parental preconception smoking is a risk factor for childhood cancers in general. Statistically significant point estimates of risks just above unity can be accounted for by the potential confounders. There are no statistically significant trends with increasing number of cigarettes smoked. Failure to replicate the findings of two nationwide and multiregional case–control studies in the UK (Sorahan *et al*, 1995,1997a,1997b,^2001^), which found significant associations with paternal preconception smoking, may be due, in part, to a higher smoking prevalence, and a larger proportion of heavy smokers in those earlier studies. More importantly, perhaps, the present study is more likely to be subject to reporting bias because of increased public awareness of adverse effects of smoking and blinding parents with respect to the study hypothesis regarding smoking was impracticable.

It is noteworthy that the analyses of data on children dying of cancer between 1953 and 1976, which showed significant trends with paternal smoking, showed no significant association with maternal smoking after allowing for paternal smoking (Sorahan *et al*, 1995,1997a,1997b). In these earlier studies the reliability of self-reported smoking in mothers was suggested by analyses of birth weights, which showed lower birth weights in children of smoking mothers. These data were collected at a time when there was little pressure on mothers to stop smoking during pregnancy and therefore less liability to bias.

The adjusted analyses in Table 4 show statistically significant negative trends for maternal smoking during pregnancy for most diagnostic groups. A protective effect for these types does not seem biologically plausible here, and it is more likely that these trends reflect an underestimation of the amount smoked by case mothers during the index pregnancy.

Birth weights of index children reported by mothers at interview were analysed in cases and controls for trend with reported numbers of cigarettes smoked during the second trimester of pregnancy. In controls, there was a trend of monotonic decrease in birth weights with numbers of cigarettes smoked (differences in mean birth weights from nonsmokers were –144.9, −237.8, −240.5, −247.1 and –252.9 g for 1–9, 10–19, 20–29, 30–39 and 40+ cpd, respectively). However, no such trend was observed among the cases (differences from nonsmokers were –209.2, −222.7, −245.6, +70.5 and –386.1 g for 1–9, 10–19, 20–29, 30–39 and 40+ cpd, respectively). These results would tend to indicate inaccurate reporting of smoking by case mothers, but they should be interpreted with caution, given the maternal source of the birth weight data. Furthermore, unusual birth weight distributions have been reported for certain childhood cancers (Yeazel *et al*, 1997), which also complicates interpretation.

Participating controls lived in more affluent areas than the originally selected controls and the cases (Law *et al*, 2002). This bias could not explain the observed case–control difference in reported maternal smoking since the risk should move in the opposite direction. Since smoking is such a well-known risk factor for cancer and for unfavourable pregnancy outcomes (e.g. low birth weight), the possibility of guilt feelings leading to under-reporting, especially in case mothers, must be considered (Heller *et al*, 1998). This is suggested by the smoking prevalence at various time points (Table 7

**Table 7:** Prevalence (%) of maternal cigarette smoking in cases and controls

	**Time point**			
	**Preconception**	**1st trimester**	**2nd trimester**	**3rd trimester**
Cases	33.7	25.7	22.4	22.4
Controls	33.7	26.9	24.2	23.9

). Slightly more case than control mothers reported giving up smoking in pregnancy. In addition, it is known that some smokers deny having smoked, denial rates varying with the phrasing of questions and whether medical advice against smoking had been given (Lee, 1998).

A separate study on reactions of parents before, during and after interview was independently carried out by sending questionnaires to a subset of 371 cases and 380 controls as soon as the interview was completed (Jenkinson *et al*, 2001). The results lend support to the proposition that the pattern shown in Table 7 is because of reporting bias in the case mothers since differential reactions between case and control mothers were found. Case mothers felt more difficulty than controls (20 *vs* 11%, *P*=0.02) when responding to questions about smoking in pregnancy. In contrast, case and control mothers reported a similar level of difficulty over questions about employment history (11 *vs.* 11%, *P*=0.91). Occupations are not generally perceived to be associated with the risk of childhood cancer (McKinney *et al*, 2002).

While smoking during pregnancy is well-known to have adverse effects on the fetus, the possible mutational effects of tobacco products on germ cells would not generally be understood. It is unlikely therefore that responses to questions about preconceptional smoking would be influenced in the same way, so these data are likely to be more reliable.

The only clear positive association to emerge from these analyses is between maternal preconceptional smoking and hepatoblastoma. This may be a chance finding arising because of multiple testing. However, detailed results support the finding, and potential confounders (deprivation, maternal/paternal ages at diagnosis of the index child) have not accounted for it. Trends with numbers of cigarettes smoked approached statistical significance (*P*=0.058). Smoking by both parents increased the risk more than four-fold (*P*=0.003). Furthermore, maternal smoking was strongly associated with cases diagnosed above the median age, suggesting specificity in the timing of a putative carcinogenic event. Since a chance association would predict a random distribution among subgroups analysed, the results suggest that parental smoking might increase the risk of hepatoblastoma. Alternatively, other unknown risk factors associated with parental smoking may explain the results. If causal, the most plausible explanation would be a transplacental carcinogenic effect of tobacco products, including passive smoking from the father's cigarettes affecting the embryonal and/or fetal liver rather than maternal germ cells. We did not collect information on paternal smoking during the pregnancy and analyses of reported maternal smoking during pregnancy do not support this explanation. However, for reasons mentioned above regarding probable under-reporting of maternal smoking, preconception smoking can be used as a proxy for pregnancy smoking.

Tobacco-specific carcinogens can cross the placental barrier to reach the fetal liver and potentially lead to mutations in oncogenes or tumour suppressor genes. Fifty-five proven carcinogens have been found in cigarettes, including polycyclic aromatic hydrocarbons, *N*-nitrosamines, aromatic amines, heterocyclic aromatic amines, aldehydes, aza-arenes, other organic compounds and inorganic compounds (Hecht, 1999). Of these, *N*-nitroso compounds are the most likely candidate transplacental liver carcinogens (Anderson *et al*, 1989; Correa *et al*, 1990; Beebe *et al*, 1993; Schuller *et al*, 1993,^1994^; Hecht, 1998). These compounds can cross the human placenta, and their metabolites have been found both in the urine and bound to the fetal haemoglobin of newborns whose mothers smoked cigarettes (Coghlin *et al*, 1991; Lackmann *et al*, 1999) and in the fetus in early pregnancy (Milunsky *et al*, 2000). Cord blood T lymphocytes from newborns has revealed an increased level of mutations (deletions in infants of mothers who smoked (Finette *et al*, 1997)). The liver is a target organ for transplacental carcinogenesis in experimental animals and, indeed, the enzymes necessary for their bioactivation are more active in human than in animal fetal liver (Everson, 1980).

For adults, carcinogens in cigarettes mainly target the first exposed organ, the lung, (Hecht, 1999), but in the fetus, the first exposed organ may be the liver, so a role for parental smoking during pregnancy may be biologically plausible in childhood hepatoblastoma. However, although a causal association with hepatoblastoma should be considered, given the number of comparisons made the apparent association could well be due to chance.

In conclusion, no statistically significant positive associations between parental smoking behaviour and major groups of cancers in children were identified by the UKCCS. However, causal associations cannot be ruled out. If there is under-reporting of smoking in the case parents, the true effects would be expected to be higher than observed in the present study. To resolve this question, new approaches are required and the integration of biomarkers for genotypes and phenotypes specific to tobacco products would be helpful in pursuing the possible role of parental smoking in the aetiology of childhood cancer.

## APPENDIX

Management Committee – KK Cheng, Central region; NE Day, East Anglia region; R Cartwright, A Craft, North East region; JM Birch, OB Eden, North West region; PA McKinney, Scotland; J Peto, South East region; V Beral, E Roman, South Midlands region; P Elwood, South Wales region; FE Alexander, South West region; CED Chilvers, Trent region; R Doll, Epidemiological Studies Unit, University of Oxford, Oxford; GM Taylor, Immunogenetics Laboratory, University of Manchester, Manchester; M Greaves, Leukaemia Research Fund Centre, Institute of Cancer Research; D Goodhead, Radiation and Genome Stability Unit, Medical Research Council, Harwell; FA Fry, National Radiological Protection Board; G Adams, UK Coordinating Committee for cancer research.

Regional Investigators – KK Cheng, E Gilman, Central region; NE Day, J Skinner, D Williams, East Anglia region; R Cartwright, A Craft, North East region; JM Birch, OB Eden, North West region; PA McKinney, Scotland; J Deacon, J Peto, South East region; V Beral, E Roman, South Midlands region; P Elwood, South Wales region; FE Alexander, M Mott, South West region; CED Chilvers, K Muir, Trent region.

Data Processing Group – GR Law, J Simpson, E Roman.
